# Barriers to Dissemination of Local Health Data Faced by US State Agencies: Survey Study of Behavioral Risk Factor Surveillance System Coordinators

**DOI:** 10.2196/16750

**Published:** 2021-07-13

**Authors:** Manik Ahuja, Robert Aseltine Jr

**Affiliations:** 1 Department of Health Services Management and Policy College of Public Health East Tennessee State University Johnson City, TN United States; 2 Center for Population Health UConn Health Farmington, CT United States

**Keywords:** web-based data query systems, WDQS, health data, population health, dissemination of local health data

## Abstract

**Background:**

Advances in information technology have paved the way to facilitate accessibility to population-level health data through web-based data query systems (WDQSs). Despite these advances in technology, US state agencies face many challenges related to the dissemination of their local health data. It is essential for the public to have access to high-quality data that are easy to interpret, reliable, and trusted. These challenges have been at the forefront throughout the COVID-19 pandemic.

**Objective:**

The purpose of this study is to identify the most significant challenges faced by state agencies, from the perspective of the Behavioral Risk Factor Surveillance System (BRFSS) coordinator from each state, and to assess if the coordinators from states with a WDQS perceive these challenges differently.

**Methods:**

We surveyed BRFSS coordinators (N=43) across all 50 US states and the District of Columbia. We surveyed the participants about contextual factors and asked them to rate system aspects and challenges they faced with their health data system on a Likert scale. We used two-sample *t* tests to compare the means of the ratings by participants from states with and without a WDQS.

**Results:**

Overall, 41/43 states (95%) make health data available over the internet, while 65% (28/43) employ a WDQS. States with a WDQS reported greater challenges (*P*=.01) related to the cost of hardware and software (mean score 3.44/4, 95% CI 3.09-3.78) than states without a WDQS (mean score 2.63/4, 95% CI 2.25-3.00). The system aspect of standardization of vocabulary scored more favorably (*P*=.01) in states with a WDQS (mean score 3.32/5, 95% CI 2.94-3.69) than in states without a WDQS (mean score 2.85/5, 95% CI 2.47-3.22).

**Conclusions:**

Securing of adequate resources and commitment to standardization are vital in the dissemination of local-level health data. Factors such as receiving data in a timely manner, privacy, and political opposition are less significant barriers than anticipated.

## Introduction

It is widely acknowledged in the public health field that progress in information technology has paved the way for exciting opportunities to disseminate local level health data more efficiently [1,2]. The growth of the internet, mobile technologies, artificial intelligence, and other technological advances have enabled health information to become more easily accessible and widely available to a broader population [2-4]. There has been growing enthusiasm for the application of big data and its utility in public health, particularly for population-level health data [5]. Access to high quality population-level health data is essential for public health, as it informs us of disease tracking, health problems, and health surveillance at the subpopulation level [6,7]. Health informatics has enabled public health practitioners to assess public and population health information by accurately combining data from a wide range of disparate sources [7]. Despite the vast advances in technology, there are many challenges associated with availability of high-quality population-level health data [7,8]. These problems have been even further exacerbated during the COVID-19 pandemic, as there is a lack of consistency in the data and their reporting [9,10].

As each US state is responsible for its own health surveillance, decisions regarding technology implementation have varied from state to state [11]. One popular option is for state agencies to design and develop dynamic web-based data query systems (WDQSs), which allow users to customize data queries by choosing data sets, variables, measures, and the format for presenting query results [12]. There are many benefits to WDQSs, as state agencies are able to respond to data requests in a timely manner, provide data to a broader population, and contribute to the development of community health assessments and policy decisions [13]. Another popular option is for states to make static reports available to the public on the World Wide Web. Static reports are generally manually compiled by staff and are available in formats such as PDF, Microsoft Excel, and HTML. Static reports limit user choice to precalculated statistics and do not allow users to choose parameters for a query [14]. Given the advances in information technology, deployment of these outdated technologies, which can be difficult to use and plagued by missing or incomplete data, by US states is unfortunate [15]. In the private sector, industries such as finance have been successful in maximizing the potential of the internet, as changes in stock prices are made available within seconds to end users.

WDQSs were first implemented in the late 1990s, as states developed systems in which queries could be specified and results returned on the World Wide Web without requiring any additional software [12]. At the time, strong efforts were being made to achieve data liberation and use of open-source information technology solutions and collaboration to promote public health [15]. Collaboration has contributed to states sharing developments, ideas, and knowledge to meet a variety of public health assessment needs [16-18]. To reduce the cost burden, there has been a push toward open-source software, which costs little or no money to procure [19]. Open-source code can be easily shared, and its key benefits include free redistribution, inclusion of source code, easy modification of the code, and lack of need for an additional license [20]. For example, in 2000, Utah’s legacy Indicator Based Information System–Public Health (IBIS-PH) was developed using open-source code, and states such as New Mexico and Kentucky adopted the system within the first few years of its development. However, over the last decade, efforts toward WDQS implementation have stalled, and progress has slowed. Public health is at risk of falling behind from a technological standpoint [21]. Despite the significant advances in technology, including faster processors, improved bandwidth, and lower cost of storage, state agencies commonly face data sharing barriers between organizations within their state [21]. The purpose of this study was to investigate the barriers faced by state agencies to the implementation of WDQSs from the perspectives of Behavioral Risk Factor Surveillance System (BRFSS) coordinators from each state. We aimed to understand how challenges and perceptions of systems differ between states that have implemented a WDQS and states that have not. We hypothesized that the perceptions and challenges reported by the BRFSS coordinators would vary across states because of the coordinators’ significance in releasing health data and their role in technology decisions in their states [14].

## Methods

### Study Design and Sampling

We designed and administered a web-based questionnaire to BRFSS coordinators from all 50 states and the District of Columbia (n=51). The BRFSS, established in 1984, is a health-related telephone survey that collects state data from US residents in all 50 states regarding their health-related risk behaviors, chronic health conditions, and use of preventive services. The BRFSS coordinator in each state is responsible for gathering information about health behaviors and is responsible for the management and oversight of the BRFSS survey [22,23]. First, we queried the coordinators on how states disseminate their data. In January 2015, each prospective participant was sent an invitation letter by US Mail. We found the names and contact information of all the BRFSS coordinators on the US Centers for Disease Control and Prevention website [24]. The letter provided details of the study and indicated that participation was confidential, as the results would not be disclosed to anyone except the study staff. The letter specified that the coordinators’ participation was voluntary and that they could withdraw at any point of the survey. Each participant was sent a follow-up email containing a link to the survey with a secure user name and password within 1-2 weeks upon receipt of the letter.

We queried the participants on the basic characteristics of their data dissemination, including how their health data are disseminated, which technology is used, and which types of data are available. We asked, “Does your state present health data over the internet?” and participants answered yes or no. We also queried if their state presents data using an interactive WBQS, which types of data are publicly available (eg, BRFSS, births, deaths), and finally, which path they took to develop their software (eg, in-house, adopted from an outside vendor). We also asked participants to rate the level of challenges they face regarding the dissemination of their data on a 4-point Likert scale (1, not at all challenging; 2, not very challenging; 3, somewhat challenging; 4, very challenging). A Likert scale is a set of statements (items) offered for a hypothetical situation under study, in which participants are asked to show their level of agreement (eg, strongly agree, somewhat agree). We presented 2 to 4 questions for three different categories: cost, staffing and support, and other challenges. We then asked the coordinators how they would rate various system-related aspects of their health data systems. They were presented a list of items and rated each item on a 5-point Likert scale (1, poor; 2, fair; 3, good; 4, very good; 5, excellent). For the system aspects, 3 to 4 questions were presented for each category, including website performance, data quality, and accessibility and support.

### Data Analysis

We obtained descriptive statistics and profile characteristics for the participants from each state. We also compared the means of the ratings of participants from states that have implemented a WDQS and from states that have not implemented a WDQS. The means were compared using two-sample *t* tests between states with and without a WDQS, with *P*<.05 used as the level of significance.

### Ethics Approval and Consent to Participate

Ethics approval for the study protocol was received from the Human Subjects Protection Office at the University of Connecticut Health Center.

## Results

Profile characteristics at the state level can be found in Table 1. A total of 43 of the 51 coordinators completed the survey, for an overall response rate of 84%. Of the 43 participants, 42 completed the web-based survey, while 1 participant completed the survey over the telephone. Of the 43 states, 41 (95%) had some form of health data available on the web, while 28 (65%) reported having implemented a WDQS. Data available included BRFSS (33/43, 77%), births (31/43, 72%), deaths (30/43, 70%), lead screening (5/43, 12%), and hospitalizations (4/43, 9%).

**Table 1:** Profile of health data characteristics at the state level (n=43).

Characteristic and responses			Value, n (%)
**Health data are made available on the internet**			
	Yes	41 (95)	
	No	2 (5)	
**Health data are made available using an interactive web-based query system**			
	Yes	28 (65)	
	No	15 (35)	
**Types of data that are publicly available^a^**			
	BRFSS^b^	33 (73)	
	Births	31 (72)	
	Deaths	30 (70)	
	Lead screening	5 (12)	
	Hospitalization	4 (9)	
**Software development path**			
	In-house	13 (37)	
	Outside vendor	8 (19)	
	Adopted from another state	6 (14)	
	Off the shelf commercial software	4 (9)	

^a^More than one response is acceptable for this question, as a state may have multiple data sources.

^b^BRFSS: Behavioral Risk Factor Surveillance System.

We report the mean ratings for the challenges faced and system aspects in Table 2. On a 4-point Likert scale, participants rated the cost of system development (mean score 3.33), consultants/vendors (mean score 3.05), and the cost of hardware/software (mean score 2.89) as the most challenging. The overall mean score for all challenges faced was 2.68 on the 4-point Likert scale. Participants reported the lack of political support (mean score 1.77) and issues with data privacy (mean 2.55) as less of a challenge. We analyzed the mean results of the reported challenges between states with and without a WDQS (Table 2). Participants from states without a WDQS reported the cost of hardware and software to be a greater challenge than those from states with a WDQS (mean score 3.55, 95% CI 3.09-3.78, vs mean score 2.63, 95% CI 2.25-3.00; *P*=.01). System aspects were rated higher (*P*=.01) for the standardization of vocabulary by participants from states with a WDQS (mean 3.32, 95% CI=2.94-3.69) versus those from states without a WDQS (mean=2.85, 95% CI 2.47-3.22).

**Table 2:** Participant ratings of challenges faced by state agencies and of system aspects (N=43).

Grouping and item				Overall mean score^a^		With WDQS^b^ (n=28), mean (95% CI)		Without WDQS (n=15), mean (95% CI)		*P* value^c^
**Challenges faced (scored on a 4-point Likert scale)**										
	**Cost**									
		Cost of system development	3.33		3.05 (2.68-3.41)		3.6 (3.28-3.91)		.06	
		Cost of hardware/software	2.89		2.63 (2.25-3.00)		3.44 (3.09-3.78)		*.01* ^d^	
		Cost of vendors/consultants	3.06		2.93 (2.44-3.41)		3.25 (2.63-3.86)		.42	
	**Staffing and support**									
		Lack of internal information technology staff	2.97		2.90 (2.37-3.42)		3.10 (2.48-3.71)		.67	
		Help desk support	2.60		2.40 (1.80-3.02)		3.00 (2.25-3.74)		.27	
		Lack of trained staff who understand the data	2.62		2.42 (1.98-2.85)		3.00 (2.49-3.50)		.12	
		Receiving data in a timely manner	2.40		2.36 (2.07-2.76)		2.42 (1.98-2.85)		.85	
	**Other challenges**									
		Privacy	2.55		2.37 (2.06-2.67)		2.9 (2.28-3.51)		.11	
		Political opposition	1.77		1.6 (1.10-2.11)		2.14 (1.47-2.80)		.23	
**System aspects (scored on a 5-point Likert scale)**										
	**Website usability**									
		User-friendliness	3.15		3.15 (2.73-3.56)		3.00 (2.69-3.32)		.64	
		Website performance	3.34		3.54 (3.15-3.93)		2.92 (2.47-3.36)		.06	
		Standardization of vocabulary	3.14		3.32 (2.94-3.69)		2.85 (2.47-3.22)		*.01*	
		End user satisfaction	3.03		3.37 (2.89-3.84)		2.40 (2.08-2.71)		.11	
	**Data quality**									
		Availability of race, gender, and other social determinants	3.91		4.05 (3.63-4.46)		3.64 (3.24-4.03)		.11	
		Quality of data	3.90		4.00 (3.62-4.37)		3.69 (3.28-4.09)		.33	
		Breadth of data	3.33		3.42 (2.96-3.87)		3.15 (2.71-3.58)		.46	
		Ability to link to multiple data sources	2.81		3.28 (2.57-3.98)		1.75 (1.26-2.24)		*.01*	
	**Accessibility and support**									
		Accessibility to researchers	3.67		3.92 (3.56-4.27)		3.15 (2.66-3.63)		.06	
		Accessibility to nonresearchers	3.58		3.76 (3.39-4.12)		3.23 (2.69-3.76)		.11	
		Timeliness of support requests	3.53		3.68 (3.28-4.07)		3.27 (2.73-3.80)		.23	

^a^The overall mean score represents the full sample.

^b^WDQS: web-based data query system.

^c^Two-sample *t* tests were used to compare the mean scores between states with and without a WDQS.

^d^Italic text indicates statistical significance at *P*<.05.

## Discussion

To our knowledge, this is the first national study to investigate barriers faced by state agencies to the dissemination of their health data using informants in key roles. The findings revealed that BRFSS coordinators rated their systems more favorably in states where a WDQS was implemented. Interestingly, despite the high cost of technology, staffing, implementation, and maintenance of technology-based systems and other factors, BRFSS coordinators from states that implemented a WDQS perceived their systems more favorably. We hypothesize that these findings are indications of a favorable assessment of the cost-benefit ratio of implementation of technology-based systems relative to low-cost health data systems. Adequate staffing and funding for state health data systems is lacking, which has impeded or slowed progress or halted data dissemination efforts in these states [25,26]. Our findings are more important than ever, given the reliance of society on trusted, reliable, and accurate public health data [27,28].

Prior research has indicated that organizations are reluctant to share their data due to organizational, technical, and political barriers [15]. In the current study, respondents reported that state agencies are generally willing to share their data and do not perceive political barriers as a significant challenge to data sharing. However, these findings should be taken with caution, as there may be bias because these perspectives were based exclusively on the experience of BRFSS coordinators, which may not be representative of that of other key stakeholders across states. Respondents also reported lack of interoperability between systems, as data may be transmitted in formats that are incompatible with the originating system. These findings are in line with prior work, in which it was reported that departments lack adequate staffing and resources to profile, “cleanse,” and manipulate these data so they are usable [15,29]. If data are not usable, they have limited utility and do not create significant opportunities for public health research. According to the latest Public Health Workforce Interests and Needs Survey (PH WINS), a nationally representative survey of the public health workforce, state agencies lack adequate trained staff who are able to handle and interpret these data [30,31]. Public health agencies are lacking workers in areas such as data-informed decision-making, health informatics, and data quality, which are essential in the dissemination of public health data [30-32].

Our findings should be interpreted with certain limitations in mind. First, the results may not be generalizable beyond the perspective of the BRFSS coordinator in each state. As each state may have multiple stakeholders who have a vested interest in the WDQS, the opinion of the BRFSS coordinator may not be representative of the consensus from that state. Secondly, our study may reflect bias, as BRFSS coordinators in states with a WDQS may rate their systems higher due to the additional investments states have made in this technology. Third, because the study includes a small number of participants, there is insufficient statistical power to detect small differences in ratings among states with and without a WDQS. Fourth, as BRFSS coordinators from 7/51 states (14%) did not respond to the survey, there may be systematic bias related to the missing information from these states. The reasons that the BRFSS coordinators from those states refused to participate are also unknown. Fifth, questions may be interpreted differently from one state to the next. Web-based expertise and technical maturity may also vary from one state to another, depending on their experience. Finally, although measures such as quality, timeliness, satisfaction, and access were assessed for multiple constructs, their definitions were not presented in the survey. Respondents may have interpreted these measures differently, potentially resulting in bias. For example, the definition of “quality” may be perceived differently from one state to the next. Despite these limitations, the current study is, to our knowledge, the first to compare system ratings and assessments of challenges to presenting health data to the public among states with more primitive versus more advanced data systems. Directions for future research include more comprehensive efforts to evaluate the utility of WDQSs, as evidence of their usefulness and their potential impact on public health may help justify the additional expenditures required. Additionally, it is recommended that state agencies aim toward collaboration and investigate open-source software options. This model has been successful in the clinical setting. For example, open-source software has been adopted by several hospitals and clinics. A similar model can be applied for future WDQS development, as states should aim to collaborate and work toward building robust systems that are easy adoptable. In summary, it is important to design systems that facilitate access to local health data; these data provide information regarding health challenges at the subpopulation level, which will ultimately help guide future public health research. These problems have been at the forefront during the COVID-19 pandemic [33,34] and should be urgently addressed moving forward.

### Data Availability

Data and materials are available upon request.

## Abbreviations

BRFSS: Behavioral Risk Factor Surveillance System
IBIS-PH: Indicator Based Information System–Public Health
PH WINS: Public Health Workforce Interests and Needs Survey
WDQS: web-based data query system

## References

[1] Buehler James W, Centers for Disease Control and Prevention (2012). CDC's vision for public health surveillance in the 21st century. Introduction. MMWR Suppl.

[2] Ye J (2020). The role of health technology and informatics in a global public health emergency: practices and implications from the COVID-19 pandemic. JMIR Med Inform.

[3] Khan Z Faizal, Alotaibi SR (2020). Applications of artificial intelligence and big data analytics in m-health: a healthcare system perspective. J Healthc Eng.

[4] Shaban-Nejad A, Michalowski M, Buckeridge DL (2018). Health intelligence: how artificial intelligence transforms population and personalized health. NPJ Digit Med.

[5] Pastorino R, De Vito Corrado, Migliara G, Glocker K, Binenbaum I, Ricciardi W, Boccia S (2019). Benefits and challenges of Big Data in healthcare: an overview of the European initiatives. Eur J Public Health.

[6] Bakshi Salina, Schiavoni Katherine H, Carlson Lucas C, Chang Trina E, Flaster Amy O, Forester Brent P, Kronenberg Frannie R, Pu Charles T, Rowe Jack S, Terry Dellara F, Wasfy Jason H, Bartels Stephen J, Sequist Thomas D, Meyer Gregg S, Mendu Mallika L (2021). The essential role of population health during and beyond COVID-19. Am J Manag Care.

[7] Massoudi BL, Chester KG (2017). Public health, population health, and epidemiology informatics: recent research and trends in the United States. Yearb Med Inform.

[8] Budd J, Miller BS, Manning EM, Lampos V, Zhuang M, Edelstein M, Rees G, Emery VC, Stevens MM, Keegan N, Short MJ, Pillay D, Manley E, Cox IJ, Heymann D, Johnson AM, McKendry RA (2020). Digital technologies in the public-health response to COVID-19. Nat Med.

[9] Ibrahim NK (2020). Epidemiologic surveillance for controlling Covid-19 pandemic: types, challenges and implications. J Infect Public Health.

[10] Sáez Carlos, Romero N, Conejero J, García-Gómez Juan M (2021). Potential limitations in COVID-19 machine learning due to data source variability: a case study in the nCov2019 dataset. J Am Med Inform Assoc.

[11] Gjelsvik A, Buechner J (2006). States' approaches to implementing a web-based data query system. J Public Health Manag Pract.

[12] Friedman DJ, Parrish RG (2006). Characteristics, desired functionalities, and datasets of state web-based data query systems. J Public Health Manag Pract.

[13] Shah G, Leep C, Alexander D (2014). Profile-IQ: web-based data query system for local health department infrastructure and activities. J Public Heal Manag Pract.

[14] Friedman DJ (2006). Envisioning futures for state Web-based data query systems. J Public Health Manag Pract.

[15] Love D, Shah GH (2006). Reflections on organizational issues in developing, implementing, and maintaining state Web-based data query systems. J Public Health Manag Pract.

[16] Kovach K, Welter C, Seweryn S, Torres G (2019). Perceived benefits of collaboration between local health departments and schools and programs of public health: a mixed-methods study. J Public Heal Manag Pract.

[17] Valaitis RK, O’Mara L, Wong ST, MacDonald M, Murray N, Martin-Misener R, Meagher-Stewart D (2018). Strengthening primary health care through primary care and public health collaboration: the influence of intrapersonal and interpersonal factors. Prim Health Care Res Dev.

[18] Shahzad M, Upshur R, Donnelly P, Bharmal A, Wei X, Feng P, Brown AD (2019). A population-based approach to integrated healthcare delivery: a scoping review of clinical care and public health collaboration. BMC Public Health.

[19] Wheeler DA Why open source software / free software (OSS/FS, FLOSS, or FOSS)? Look at the numbers!. Challenges 2014.

[20] Aminpour F, Sadoughi F, Ahmadi M (2013). Towards the application of open source software in developing national electronic health record-narrative review article. Iran J Public Health.

[21] Vest J, Issel LM, Lee S (2014). Experience of using information systems in public health practice: findings from a qualitative study. Online J Public Health Inform.

[22] Anderson N, Marcum J (2019). Using behavioral risk factor surveillance system data as an occupational health profile: Washington State janitors, 2011 to 2017. J Occup Environ Med.

[23] Iachan R, Pierannunzi C, Healey K, Greenlund KJ, Town M (2016). National weighting of data from the Behavioral Risk Factor Surveillance System (BRFSS). BMC Med Res Methodol.

[24] BRFSS State Coordinators. US Centers for Disease Control and Prevention.

[25] McFarlane T, Dixon B, Grannis S, Gibson P (2019). Public health informatics in local and state health agencies: an update from the public health workforce interests and needs survey. J Public Health Manag Pract.

[26] Colquhoun A, Aplin L, Geary J, Goodman KJ, Hatcher J (2012). Challenges created by data dissemination and access restrictions when attempting to address community concerns: individual privacy versus public wellbeing. Int J Circumpolar Health.

[27] Kostkova P, Brewer H, de Lusignan Simon, Fottrell E, Goldacre B, Hart G, Koczan P, Knight P, Marsolier C, McKendry RA, Ross E, Sasse A, Sullivan R, Chaytor S, Stevenson O, Velho R, Tooke J (2016). Who owns the data? Open data for healthcare. Front Public Health.

[28] Edelstein M, Lee LM, Herten-Crabb A, Heymann DL, Harper DR (2018). Strengthening global public health surveillance through data and benefit sharing. Emerg Infect Dis.

[29] Shah G, Madamala K (2015). Knowing where public health is going: levels and determinants of workforce awareness of national public health trends. J Public Health Manag Pract.

[30] Bogaert K, Castrucci B, Gould E, Sellers K, Leider J, Whang C, Whitten V (2019). The public health workforce interests and needs survey (PH WINS 2017): an expanded perspective on the state health agency workforce. J Public Health Manag Pract.

[31] Yeager V, Balio C, Kronstadt J, Beitsch L (2019). The relationship between health department accreditation and workforce satisfaction, retention, and training needs. J Public Health Manag Pract.

[32] Rodriguez H, Ramirez J (2015). The work experiences of community health professionals: implications for the continued rollout of the affordable care act. J Public Health Manag Pract.

[33] Fegan G, Cheah PY (2021). Solutions to COVID-19 data sharing. Lancet Digital Health.

[34] Gao F, Tao L, Huang Y, Shu Z (2020). Management and data sharing of COVID-19 pandemic information. Biopreserv Biobank.

